# Research hotspots and trends of microRNAs in spinal cord injury: a comprehensive bibliometric analysis

**DOI:** 10.3389/fneur.2024.1406977

**Published:** 2024-05-21

**Authors:** Baoyang Hu, Yue Zhao, Chao Chen, Bin Wu, Hongbin Zhang, Bin Liu, Runquan Zheng, Fang Fang

**Affiliations:** ^1^Spinal Surgery, Tongliao People's Hospital, Tongliao, Inner Mongolia, China; ^2^Computer Network Information Center, Tongliao People's Hospital, Tongliao, Inner Mongolia, China; ^3^Bone Trauma Surgery, The 960th Hospital of the PLA Joint Logistics Support Force, Jinan, Shandong, China

**Keywords:** spinal cord injury, miRNAs, bibliometrics, inflammation, nerve

## Abstract

**Background:**

Spinal cord injury (SCI) is a nervous system disease leading to motor and sensory dysfunction below the injury level, and can result in paralysis. MicroRNAs (miRNAs) play a key role in SCI treatment, and related research provides insights for SCI diagnosis and treatment. Bibliometrics is an important tool for literature statistics and evaluation, objectively summarizing multidimensional information. This study comprehensively overviews the field through bibliometric analysis of miRNA and SCI research, providing contemporary resources for future collaboration and clinical treatment.

**Materials and methods:**

In this study, we searched the Web of Science Core Collection (WOSCC) database. After careful screening and data import, we extracted annual publications, citation counts, countries, institutions, authors, journals, highly cited articles, co-cited articles, keywords, and H-index. Bibliometrics and visualization analyses employed VOSviewer, CiteSpace, the R package “bibliometrix,” and online analytic platforms. Using Arrowsmith,^1^ we determined miRNA-SCI relationships and discussed potential miRNA mechanisms in SCI.

**Results:**

From 2008 to 2024, the number of related papers increased annually, reaching 754. The number of yearly publications remained high and entered a period of rapid development. Researchers from 50 countries/regions, 802 institutions, 278 journals, and 3,867 authors participated in the field. Currently, China has advantages in the number of national papers, citations, institutions, and authors. However, it is necessary to strengthen cooperation among different authors, institutions, and countries to promote the production of important academic achievements. The research in the field currently focuses on nerve injury, apoptosis, and gene expression. Future research directions mainly involve molecular mechanisms, clinical trials, exosomes, and inflammatory reactions.

**Conclusion:**

Overall, this study comprehensively analyzes the research status and frontier of miRNAs in SCI. A systematic summary provides a complete and intuitive understanding of the relationship between SCI and miRNAs. The presented findings establish a basis for future research and clinical application in this field.

## Introduction

Spinal cord injury (SCI) is a disease of the nervous system that can lead to motor and sensory dysfunction below the injury level, and even paralysis (1). About 250,000–500,000 people suffer from this disease annually worldwide (2, 3). The lifetime treatment cost for each SCI patient in the United States is about $500,000 to $1 million, with annual costs exceeding $7 billion (4, 5). Patients experience significant physical and psychological pain, making SCI a major societal burden. The pathophysiological development of SCI is complex, involving cell death, axonal injury, glial scar formation, inflammation, and more (6). However, current clinical treatment effects are very limited, and there is no cure yet, making SCI a major focus of research.

MicroRNAs (miRNAs) are endogenous small single-stranded non-coding RNA molecules (18–25 nucleotides) that can bind to target messenger RNA (mRNA) molecules to interfere with translation (7, 8). As key regulatory factors of transcriptional gene expression changes in nervous system diseases, miRNAs have been widely concerned and studied by researchers (9). During SCI development, miRNA regulates neuronal plasticity, degeneration, axon regeneration, and myelin regeneration by changing gene expression (10, 11). Research shows miR-940 decreases after SCI, and miR-940 overexpression inhibits inflammation and promotes functional recovery (12). Another study showed miRNA-124 inhibits neuronal apoptosis and induces functional recovery in SCI rats (13). Clarifying miRNAs’ role in SCI pathophysiology is of great scientific significance for solving nerve regeneration and functional repair problems in patients.

Bibliometrics is an important tool for literature statistics and evaluation. By combining qualitative and quantitative analysis, this method can objectively and intuitively summarize multidimensional information (14). VOSviewer and CiteSpace are two popular bibliometric software (15, 16), which provide an overall view of basic data and dynamic trends. Research trends and hotspots in a field can be better explored and grasped by researchers through bibliometrics studies. Though there have been an increasing number of such studies with enhancement in bibliometrics visualization tools and research depth in recent years, no bibliometrics studies have focused on the role of miRNA in SCI.

Currently, a plethora of studies have explored the mechanistic relationship between miRNA and SCI, laying the foundation for clinical translation of SCI treatment. This study provides a comprehensive overview of the field through bibliometric analysis of miRNA and SCI research, enhancing overall understanding of researchers and providing contemporary resources for future collaboration and clinical treatment.

## Materials and methods

### Data sources and search strategies

**Figure 1:**
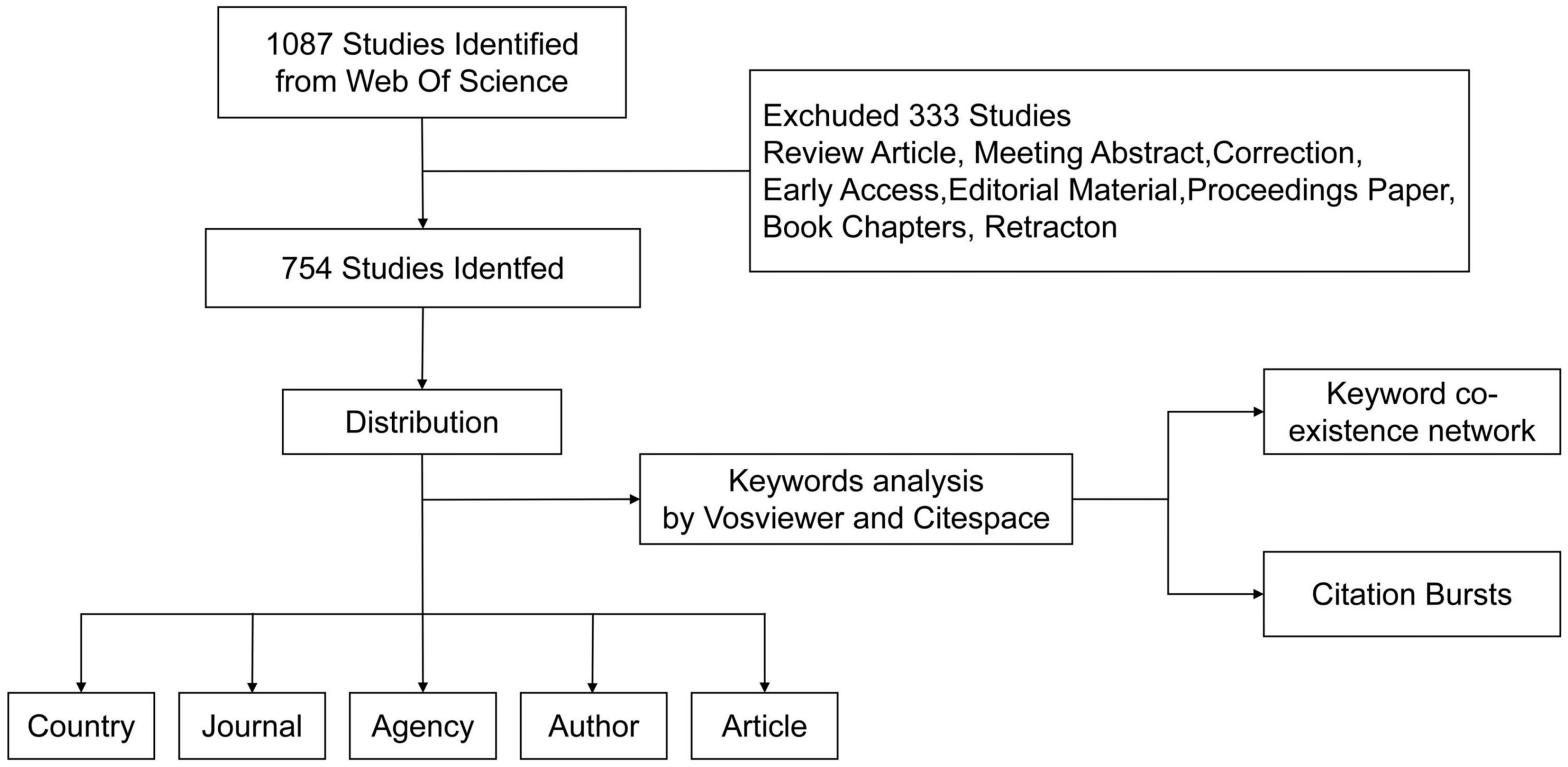
The inclusion and exclusion process of miRNAs in SCI research.

In this study, we searched the Web of Science Core Collection (WOSCC) database, which contains a large number of documents in the biomedical field. The search used the following formats: TS = (microRNA*) AND TS = (Spinal cord injury). The time span was January 1, 2008 to January 31, 2024. After carefully searching the existing literature, we screened out 1,087 potential articles. We limited the types of articles to Article and limited the language to English, finally including 754 articles (Figure 1).

### Data collection and statistics

Search results are downloaded in a “plain text file” format, with complete records and references in the WOSCC database. Data are extracted after careful screening and importing into Microsoft Excel 2016 for analysis: annual publications, citation numbers, countries, institutions, authors, journals, highly cited articles, co-cited articles, keywords, and H-index. The summarized data are imported into the online bibliometrics analysis platform,^2^ and a fitting curve is used to predict the number of published documents.

### Bibliometric analysis

To analyze the bibliometrics of SCI and miRNA, we visually analyzed selected data. These data clarified research trends, influence, and distribution. This paper used VOSviewer software for visual analysis of authors, keywords, keyword co-occurrence timeline, density maps, journals, highly cited literature, and co-cited literature. CiteSpace software was used for detailed visualization of countries/regions cooperation network map, institutions cooperation network map, strongest citation burst, keyword clustering, journal dual graph overlay, etc. The node size represents the number of nodes; the lines between nodes represent cooperation between institutions, with thicker lines indicating closer relationships. From these results, inferences about relationships between research hotspots and frontiers were made.

### Analysis of the potential mechanisms based on the Arrowsmith project

Arrowsmith (see text footnote 1) was utilized to identify relationships between miRNAs and SCI and evaluate potential miRNA mechanisms in SCI. Keywords filtered through Arrowsmith served as prediction groups, while those filtered through VOSviewer were confirmation groups. Venn diagrams were drawn for both to determine potential keywords for miRNA and SCI research. R software was used for gene ontology (GO) analysis and Kyoto Encyclopedia of Genes and Genomes (KEGG) pathway analysis of potential genes with correlation capacity ≥0.95. The STRING online database^3^ was used to create a potential gene protein–protein interaction (PPI) network, visualized with Cytoscape software. Cytoscape software also aided in screening genes within the PPI network.

## Results

### Annual publications and trends

**Figure 2:**
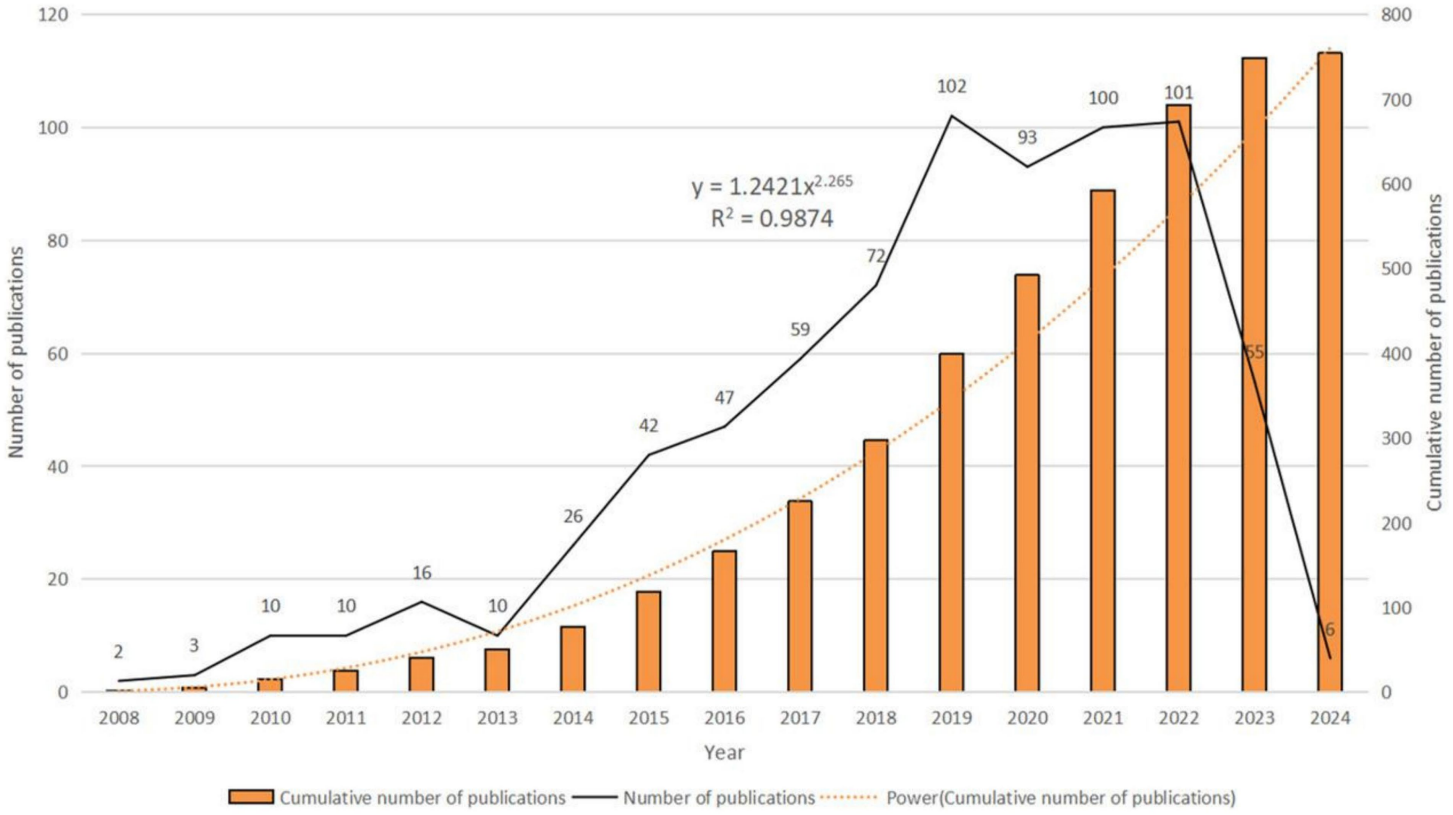
Annual publication volume and annual cumulative publication volume.

Analyzing publication trends enables understanding of the developmental speed and changing attention to miRNAs in SCI treatment (Figure 2). From 2008 to 2024, 754 papers were published. Annual publication numbers rapidly increased from 2013 to 2019, peaking in 2019 (*n* = 102). Publications in 2019–2022 were relatively flat, but decreased significantly in 2023 (*n* = 55). With only January 2024 included, it is currently the year with the fewest publications (*n* = 6). The function formula of the fitting curve is “*y* = 1.2421*x*^2.265^, *R*^2^ = 0.9874,” indicating the research growth curve in this field fits well. These results show studying miRNAs is an important direction in SCI.

### Country/region analysis and international cooperation

**Figure 3:**
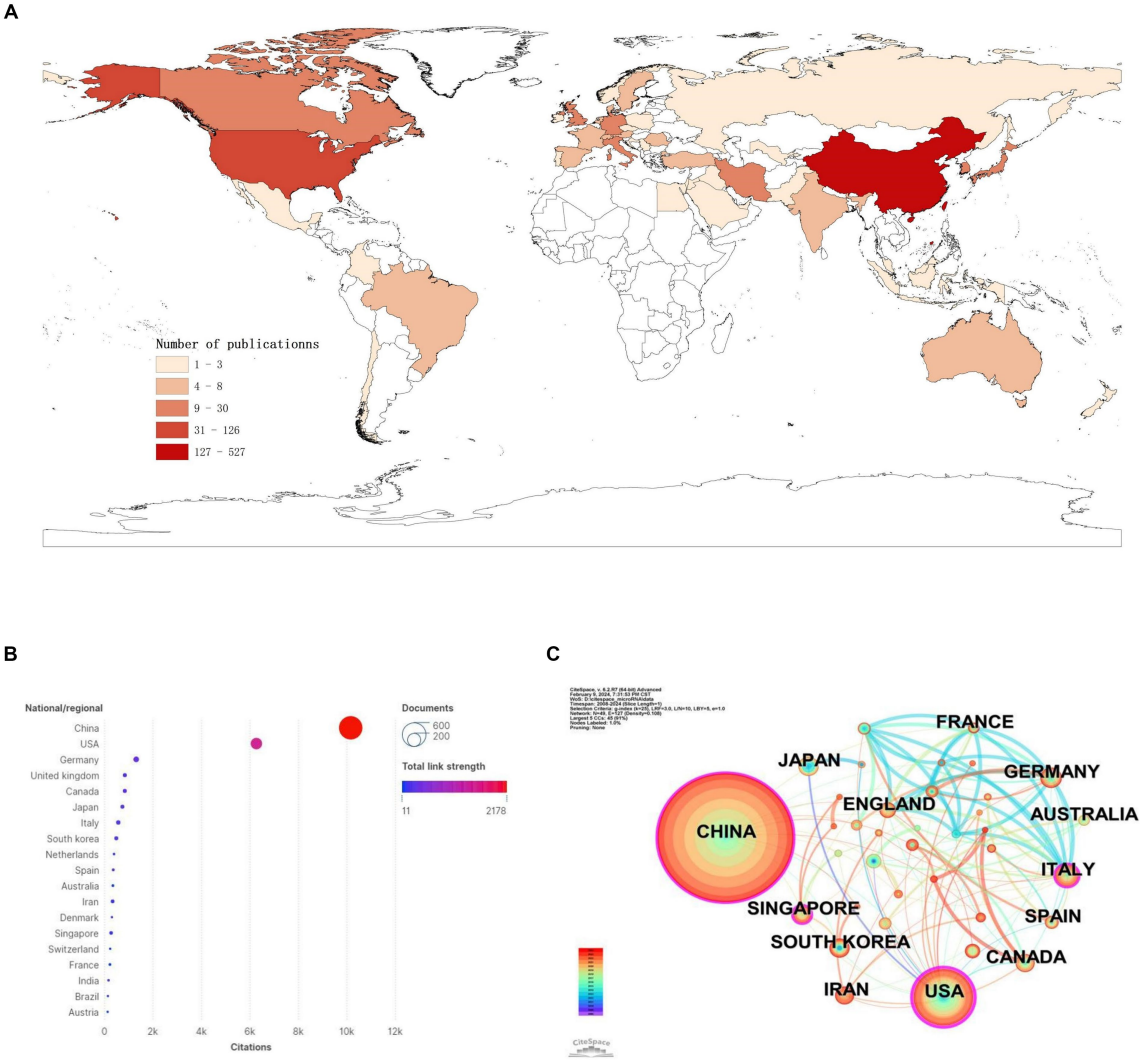
National/regional contributions of miRNAs and SCI-related research. **(A)** Comparative map of the cumulative number of papers published in each Countries/regions. **(B)** Countries/regions cited frequency bubble map. **(C)** Cooperation networks in countries/regions around the world.

We found that a total of 50 countries/regions have published literature on the role of miRNAs in SCI, with China being the most prominent country, accounting for the largest number of publications (*n* = 527; 59.48%) and citations (*n* = 10,155; 40.32%) (Figures 3A,B; Table 1). With the globalization of knowledge and technology, international cooperation is increasingly close, and the United States plays an important role in the cooperation network. European countries have the closest cooperation (Figure 3C). Overall, although China has the largest number of publications, it still lacks international cooperation and needs to strengthen exchanges in the future.

**Table 1:** Top 10 countries/regions in terms of the number of documents and citations on miRNAs in SCI.

Rank	Country	Documents(%)	Rank	Country	Citations (%)
1	China	527 (59.48)	1	China	10,155 (40.32)
2	United States	126 (14.22)	2	United States	6,264 (24.87)
3	Germany	30 (3.38)	3	Germany	1,307 (5.19)
4	Italy	18 (2.03)	4	United kingdom	838 (3.32)
5	Canada	15 (1.69)	5	Canada	836 (3.31)
6	Japan	15 (1.69)	6	Japan	738 (2.93)
7	South Korea	15 (1.69)	7	Italy	567 (2.25)
8	United Kingdom	15 (1.69)	8	South Korea	485 (1.92)
9	Iran	13 (1.46)	9	Netherlands	391 (1.55)
10	Singapore	12 (1.35)	10	Spain	360 (1.42)

### Institutional contribution

**Figure 4:**
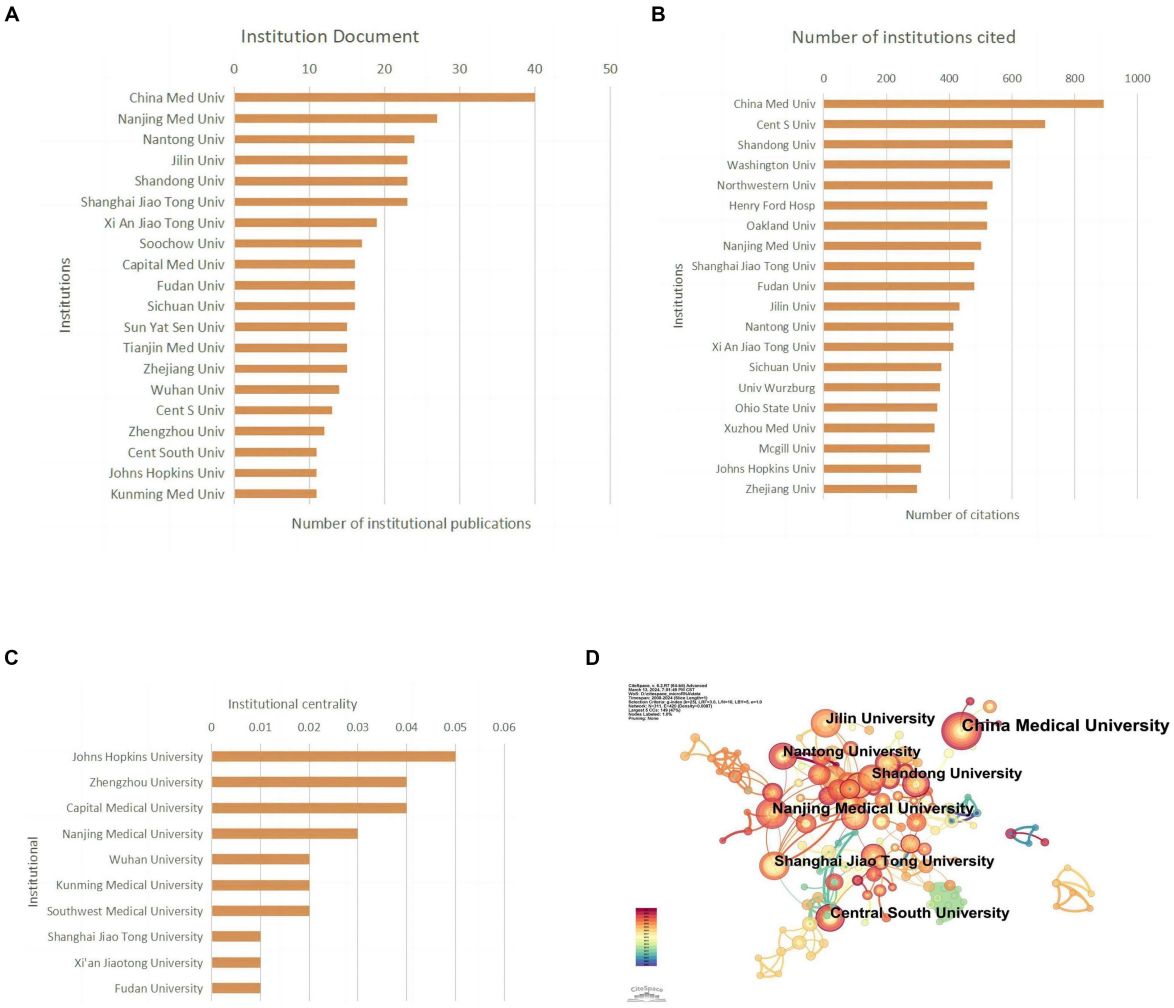
Institutional contribution on miRNAs in SCI research. **(A)** Top 10 institutions by publication volume. **(B)** Top 10 most cited institutions. **(C)** Top 10 intermediary centrality value of research institutions. **(D)** A visual network map of research institution contributions and collaborations.

Papers in this field have been published by 802 institutions. Most of the top 10 universities in publication count and citation count are from China. China Medical University published the most articles (*n* = 40; 2.67%) and received the most citations (*n* = 891; 2.26%), ranking first in both categories (Figures 4A,B; Table 2). Johns Hopkins University ranks first in centrality (coefficient = 0.05), while Chinese research institutions rank relatively high, showing that Chinese universities have made remarkable achievements and great influence in the field of miRNAs related to SCI, but there is still room for improvement (Figure 4C). Nevertheless, analyzing the scale and degree of inter-institutional collaboration reveals that institutions with more publications did not extensively collaborate with each other (Figure 4D).

**Table 2:** Top 10 institutions in terms of the number of documents and citations on miRNAs in SCI.

Rank	Institution (*n* = 730)	Documents (%)	Rank	Institution (*n* = 730)	Citations (%)
1	China Med Univ	40 (2.67)	1	China Med Univ	891 (2.26)
2	Nanjing Med Univ	27 (1.80)	2	Cent S Univ	704 (1.79)
3	Nantong Univ	24 (1.60)	3	Shandong Univ	601 (1.52)
4	Shandong Univ	23 (1.53)	4	Washington Univ	593 (1.50)
5	Shanghai Jiao Tong Univ	23 (1.53)	5	Northwestern Univ	538 (1.36)
6	Jilin Univ	23 (1.53)	6	Henry Ford Hosp	521 (1.32)
7	Xi An Jiao Tong Univ	19 (1.26)	7	Oakland Univ	521 (1.32)
8	Soochow Univ	17 (1.13)	8	Nanjing Med Univ	502 (1.27)
9	Fudan Univ	16 (1.06)	9	Shanghai Jiao Tong Univ	480 (1.22)
10	Sichuan Univ	16 (1.06)	10	Fudan Univ	480 (1.22)

### Journal analysis

**Figure 5:**
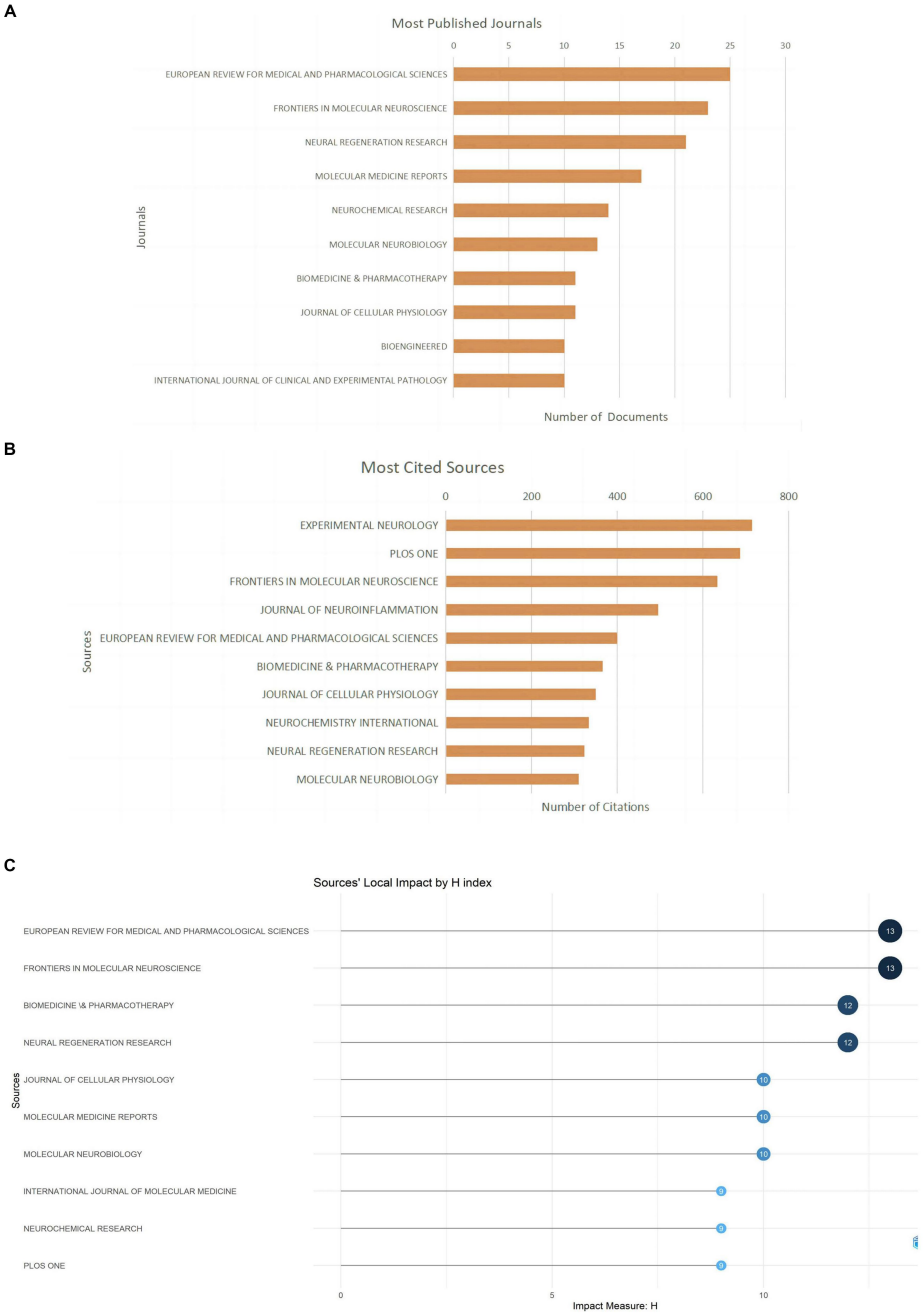

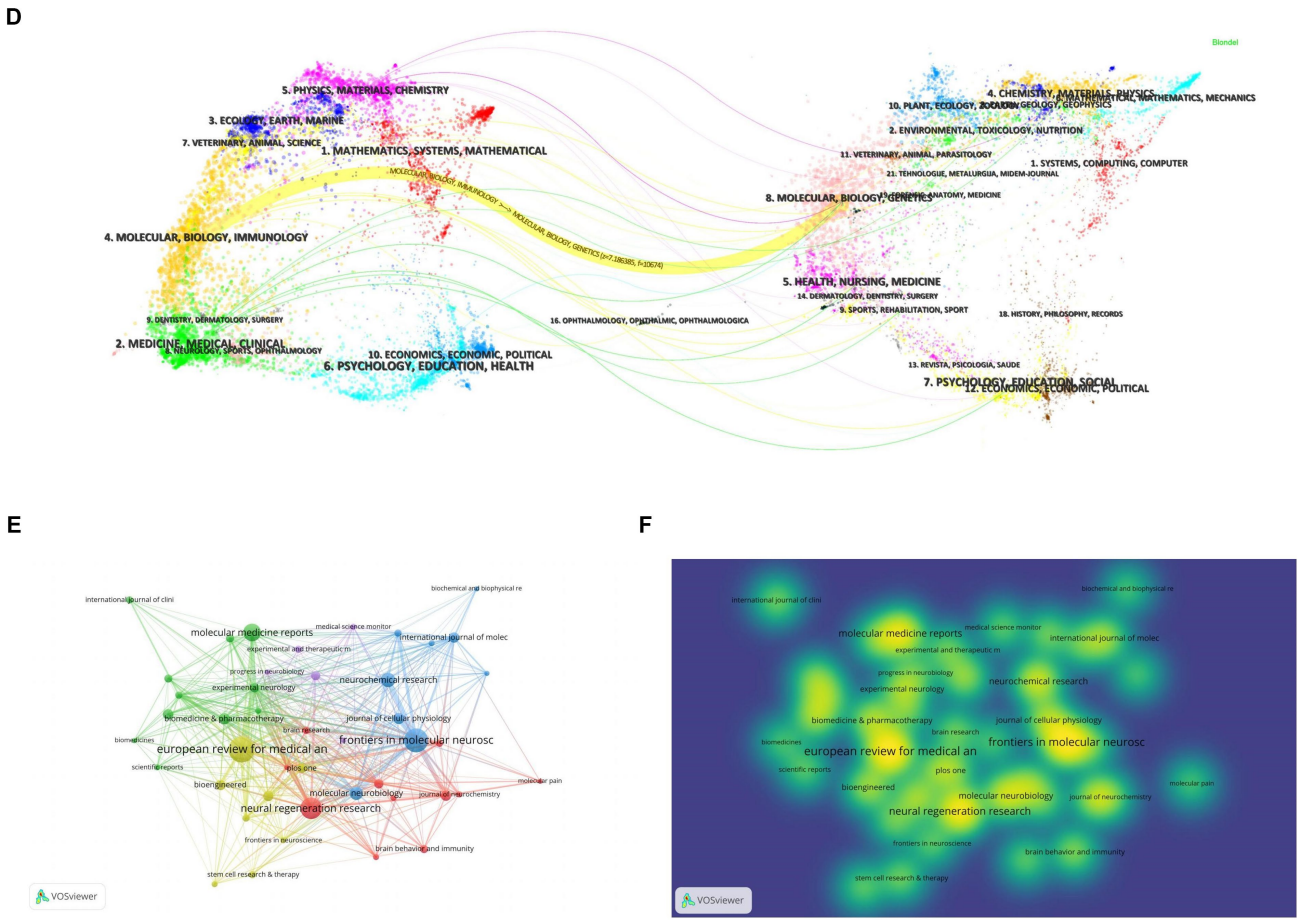
Journal characteristics of miRNAs and SCI-related research. **(A)** Top 10 journals by publication volume. **(B)** Top 10 most cited journals. **(C)** Top 10 journal H-index. **(D)** The dual-map overlay of journals in WOSCC. **(E)** Visual network map of core journals. **(F)** Visual density view of the core journals.

Related literature was published in 278 journals, with the most published article in European Review for Medical and Pharmacological Sciences (*n* = 25; 6.4%), the most cited journal in Experimental Neurology (*n* = 715; 6.9%) (Figures 5A,B; Table 3), and the highest H-index in European Review for Medical and Pharmacological Sciences (Figure 5C). Considering journal classification and influence factors, research quality needs further improvement. Analysis of superimposed journal graphs shows yellow bars indicate articles in molecular/biology/immunology journals often cite articles in molecular/biology/genetics journals (Figure 5D). Per cooperative network analysis, journals fell into four clusters, with the large node/density of European Review for Medical and Pharmacological Sciences, Frontiers in Molecular Neuroscience, and Neural Regeneration Research indicating these journals’ important roles in the field (Figures 5E,F).

**Table 3:** Top10 journals in terms of the number of documents and citations on miRNAs in SCI.

Rank	Journals (*n* = 44)	Documents (%)	Rank	Journals (*n* = 44)	Citations (%)
1	European Review for Medical and Pharmacological Sciences	25 (6.4)	1	Experimental Neurology	715 (6.9)
2	Frontiers in Molecular Neuroscience	23 (5.8)	2	Plos One	687 (6.7)
3	Neural Regeneration Research	21 (5.3)	3	Frontiers in Molecular Neuroscience	634 (6.1)
4	Molecular Medicine Reports	17 (4.3)	4	Journal of Neuroinflammation	496 (4.8)
5	Neurochemical Research	14 (3.5)	5	European Review for Medical and Pharmacological Sciences	400 (3.9)
6	Molecular Neurobiology	13 (3.3)	6	Biomedicine & Pharmacotherapy	366 (3.5)
7	Biomedicine & Pharmacotherapy	11 (2.8)	7	Journal of Cellular Physiology	350 (3.4)
8	Journal of Cellular Physiology	11 (2.8)	8	Neurochemistry International	333 (3.2)
9	Bioengineered	10 (2.5)	9	Neural Regeneration Research	324 (3.1)
10	International Journal of Clinical and Experimental Pathology	10 (2.5)	10	Molecular Neurobiology	310 (3.0)

### Author contribution and collaboration analysis

**Figure 6:**
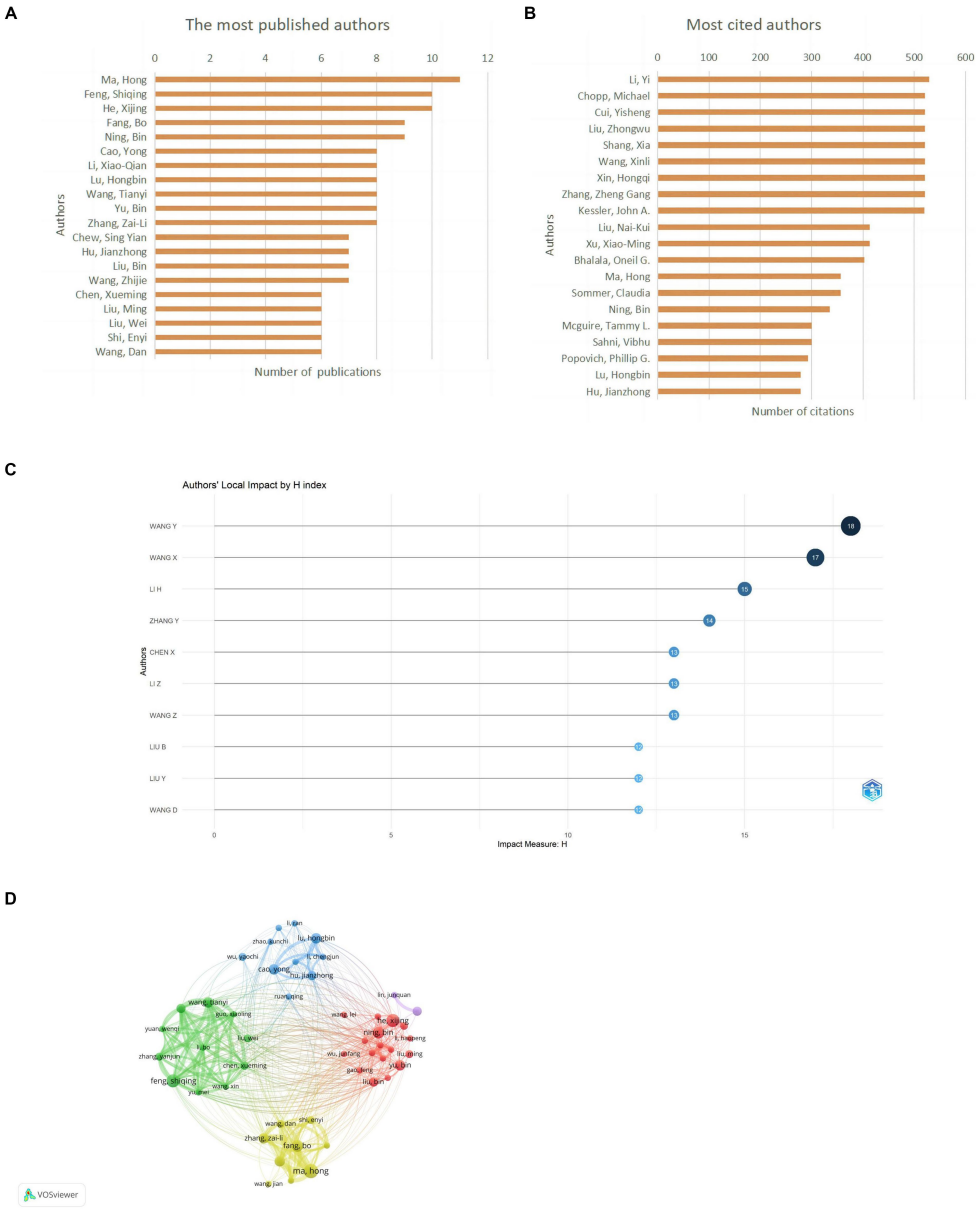
Author contribution on miRNAs in SCI research. **(A)** Top 10 authors by publication volume. **(B)** Top 10 most cited authors. **(C)** Top 10 author H-index. **(D)** A visual network map of author contributions and collaborations.

A total of 3,867 authors have published relevant papers, with Ma, H publishing the most papers (*n* = 11, 3.63%) and also being the most cited author (*n* = 357, 4.68%), indicating extensive research in this field (Figures 6A,B; Table 4). Wang, Y, with the highest H-index, is the most influential author (Figure 6C). Many academic groups are active in this field, such as Ma, H, Feng, SQ, He, XJ, Lu, HB, etc. Authors within groups collaborate closely, but inter-group collaboration is insufficient (Figure 6D).

**Table 4:** Top 10 authors in terms of the number of documents and citations on miRNAs in SCI.

Rank	Author (*n* = 3,867)	Documents (%)	Total link strength	Rank	Author (*n* = 3,867)	Citations (%)	Total link strength
1	Ma, Hong	11 (3.63)	189	1	Ma, Hong	357 (4.68)	189
2	Feng, Shiqing	10 (3.30)	160	2	Ning, Bin	335 (4.39)	111
3	He, Xijing	10 (3.30)	44	3	Lu, Hongbin	279 (3.65)	89
4	Fang, Bo	9 (2.97)	179	4	Hu, Jianzhong	279 (3.65)	80
5	Ning, Bin	9 (2.97)	111	5	Li, Xiao-Qian	270 (3.54)	163
6	Cao, Yong	8 (2.64)	80	6	Fang, Bo	268 (3.51)	179
7	Li, Xiao-Qian	8 (2.64)	163	7	He, Xijing	228 (2.99)	44
8	Lu, Hongbin	8 (2.64)	89	8	Maza, Rodrigo M.	224 (2.93)	100
9	Wang, Tianyi	8 (2.64)	186	9	Munoz-Galdeano, Teresa	224 (2.93)	100
10	Yu, Bin	8 (2.64)	65	10	Nieto-Diaz, Manuel	224 (2.93)	100

### Analysis of highly cited articles and co-citation articles

**Figure 7:**
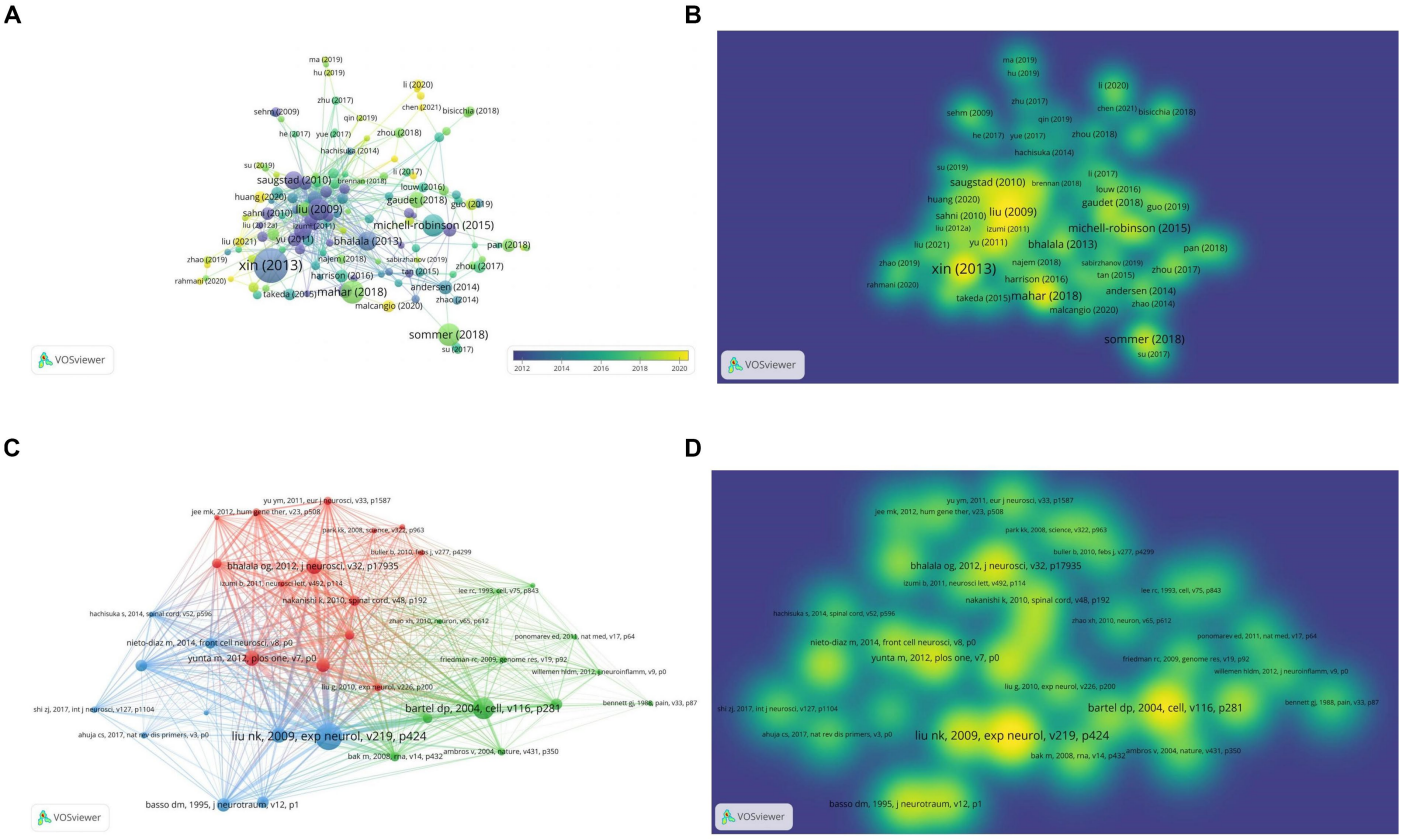
Characteristics of highly cited and co-citation articles on miRNAs and SCI. **(A)** Visual network diagram of the cited article. **(B)** Visual density view of the cited articles. **(C)** Visual network diagram of co-cited articles. **(D)** Visual density view of co-cited articles.

Table 5 shows the top 10 cited publications, led by Xin HQ’s article in Stem Cells, with 521 citations. It reported Mir-133b promotes neural plasticity and recovery post-stroke treatment via transfer of exosome-enriched particles. Liu NK’s article in Experimental Neurology, “Altered microRNA expression post-traumatic spinal cord injury,” was cited 153 times (Table 6). VOSviewer software is used to draw the visual map and visual density view of the cited documents and co-cited documents in order to obtain better visual effect. Only references with citation frequency ≥ 40 are shown in the cited chart. Xin (2013) was cited by more literatures (Figures 7A,B). Only references with citation frequency ≥ 30 are displayed in the co-cited reference map. Liu NK (2009) and Bartel Dp (2004) have attracted wide attention (Figures 7C,D).

**Table 5:** Top 10 highly cited articles on miRNAs in IDD in the WOSCC database.

Rank	Author	Title	Journal	Year	Citations
1	Xin, Hongqi	Mir-133b promotes neural plasticity and functional recovery after treatment of stroke with multipotent mesenchymal stromal cells in rats via transfer of exosome-enriched extracellular particles.	Stem Cells	2013	521
2	Mahar	Intrinsic mechanisms of neuronal axon regeneration.	Nature Reviews Neuroscience	2018	295
3	Sommer	Inflammation in the pathophysiology of neuropathic pain.	Pain	2018	278
4	Michell-robinson	Roles of microglia in brain development, tissue maintenance and repair.	Brain	2015	272
5	Liu, Nai-kui	Altered microRNA expression following traumatic spinal cord injury.	Experimental Neurology	2009	268
6	Bhalala	The emerging roles of microRNAs in CNS injuries.	Nature Reviews Neurology	2013	220
7	Saugstad	MicroRNAs as effectors of brain function with roles in ischemia and injury, neuroprotection, and neurodegeneration.	Journal of Cerebral Blood Flow and Metabolism	2010	202
8	Bhalala	MicroRNA-21 regulates astrocytic response following spinal cord injury.	Journal of Neuroscience	2012	183
9	Gaudet	MicroRNAs: roles in regulating neuroinflammation.	Neuroscientist	2018	170
10	Sun, Ping	MicroRNA-based therapeutics in central nervous system injuries.	Journal of Cerebral Blood Flow and Metabolism	2016	142

**Table 6:** Top 10 co-citation articles on miRNAs in SCI in the WOSCC database.

Rank	Author	Title	Journal	Year	Citations
1	Liu NK	Altered microRNA expression following traumatic spinal cord injury.	Experimental Neurology	2009	153
2	Bartel DP	MicroRNAs: genomics, biogenesis, mechanism, and function.	Cell	2004	124
3	Bhalala OG	MicroRNA-21 regulates astrocytic response following spinal cord injury.	Journal of Neuroscience	2012	90
4	Hu JZ	Anti-apoptotic effect of microRNA-21 after contusion spinal cord injury in rats.	Journal of Neurotrauma	2013	87
5	Yunta M	MicroRNA dysregulation in the spinal cord following traumatic injury.	PloS One	2012	87
6	Strickland ER	MicroRNA dysregulation following spinal cord contusion: implications for neural plasticity and repair.	Neuroscience	2011	77
7	Basso DM	A sensitive and reliable locomotor rating scale for open field testing in rats.	Journal of Neurotrauma	1995	76
8	Ning B	MicroRNAs in spinal cord injury: potential roles and therapeutic implications.	International Journal of Biological Sciences	2014	67
9	Livak KJ	Analysis of relative gene expression data using real-time quantitative PCR and the 2(-Delta Delta C(T)) Method.	Methods	2001	66
10	Bartel DP	MicroRNAs: target recognition and regulatory functions.	Cell	2009	65

### Keyword analysis

**Figure 8:**
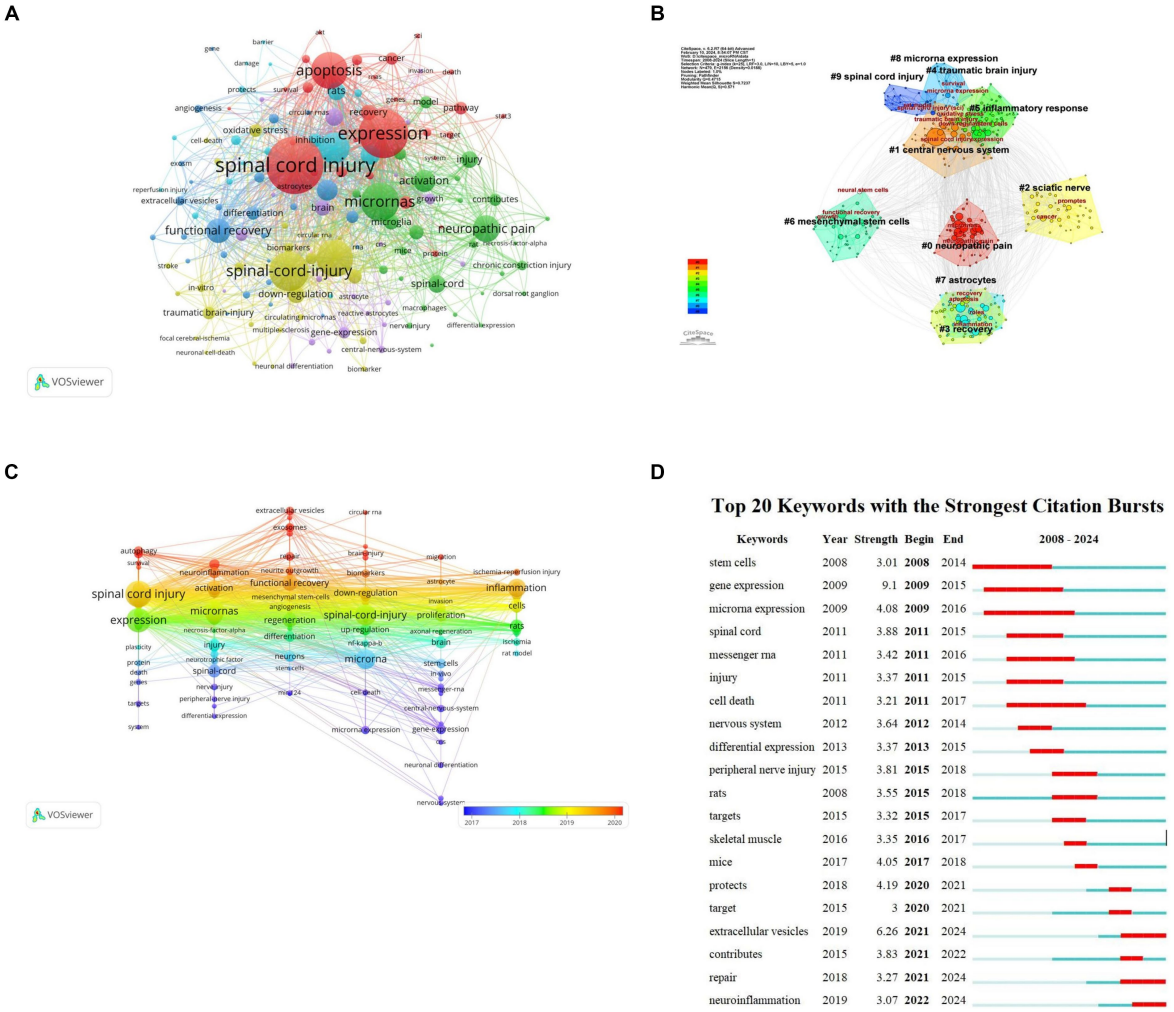
Keyword analysis of miRNAs and SCI research. **(A)** Keyword clustering network map. **(B)** Keyword clustering analysis. **(C)** Keyword co-occurrence timeline by keywords on miRNAs in SCI. **(D)** Top 20 Keyword with the strongest citation bursts on miRNAs in SCI.

Keywords are words that have a high degree of generalization of the overall research content of an article. Through co-occurrence analysis of 3,151 keywords in all literatures, it was found that research mainly focuses on SCI, expression, apoptosis, microRNAs, etc. (Figure 8A). The LLR algorithm was used to perform cluster analysis on all keywords, drawing a cluster map with a total of nine clusters. The smaller the classification number, the more keywords that classification contains. #0 neuropathic pain, #1 central nervous system; each tag is interrelated and developed, rather than existing independently (Figure 8B). According to keyword co-occurrence timeline chart of the references, this development and change can also be confirmed from 2018 to 2019, SCI related research content is the richest (Figure 8C).

Emergent words refer to keywords that appear within a short time or are used frequently reflecting a change in research direction within a field. Based on keyword intensity and duration research direction can be roughly divided into three stages: (1) 2008–2015 focusing on changes in RNA and related gene expression post spinal cord injury and stem cell therapy; (2) 2015–2018 focusing on peripheral nerve injury treatment and exploring in vivo SCI treatment in mice; and (3) 2018–2024 primarily studying extracellular vesicles for SCI repair and neuroinflammation alleviation. This development and change is also confirmed by the co-citation time chart of references. Keyword co-occurrence timeline reflects the richest SCI research content from 2018 to 2019 (Figure 8D).

### Analysis of potential mechanisms based on the Arrowsmith project

**Figure 9:**
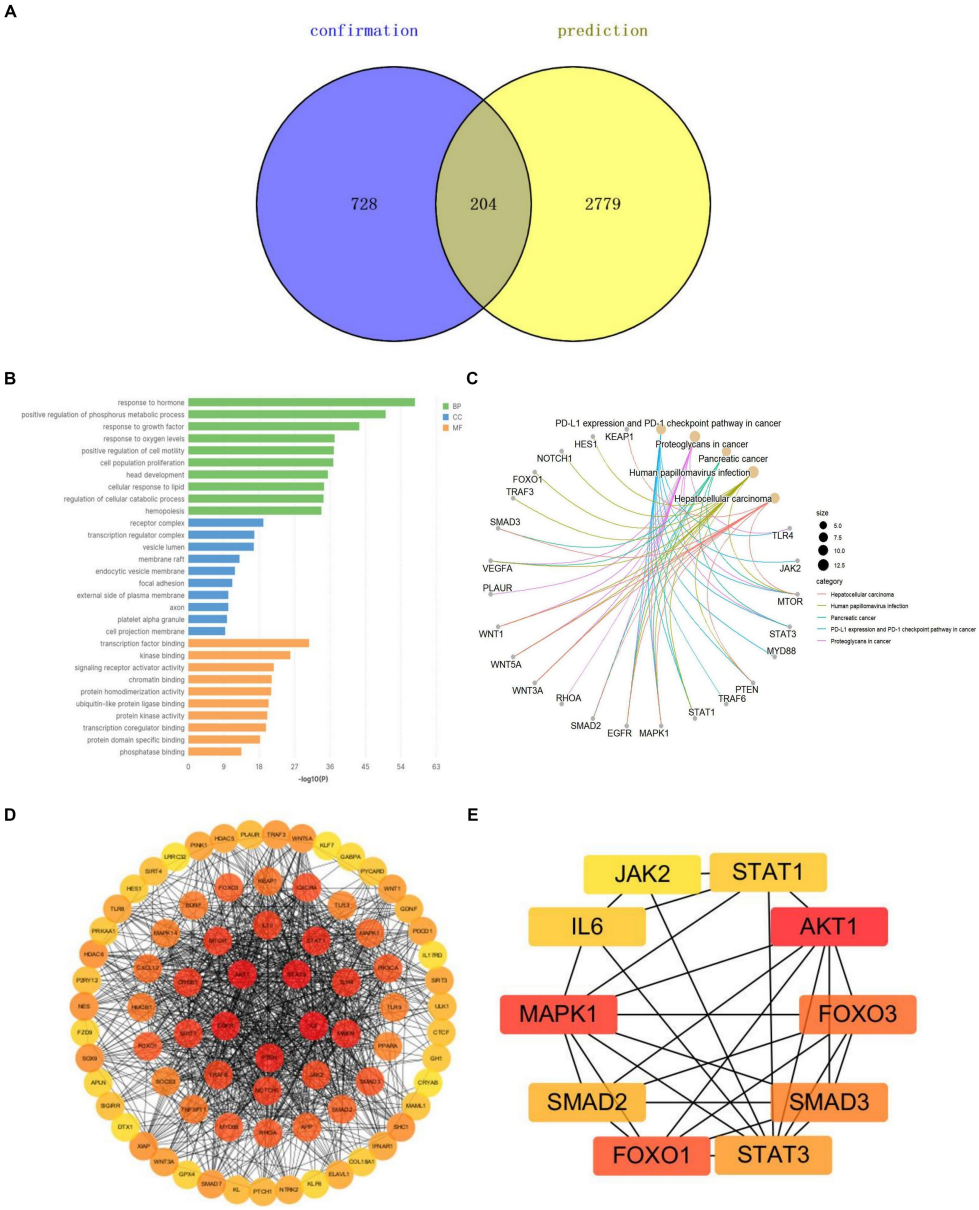
Potential keyword analysis of miRNAs and SCI research. **(A)** Venn diagrams for keywords of prediction and confirmation groups. **(B)** GO analysis of the potential key genes. **(C)** KEGG pathway enrichment analysis of the potential key genes. **(D)** PPI network of miRNA-related targets in SCI. **(E)** Hub genes of miRNA-related targets in SCI.

We selected the keywords from the Arrowsmith project as the prediction group (*n* = 2,779) and the keywords extracted by VOSviewer as the confirmation group (*n* = 728). A Venn diagram showed 204 common keywords, which can serve as potential research directions for studying miRNAs in SCI (Figure 9A). We extracted genes corresponding to these potential keywords for Gene Ontology (GO) analysis and Kyoto Encyclopedia of Genes and Genomes (KEGG) pathway enrichment analysis. The GO analysis showed biological processes (BP) focused on cell responses to hormones, oxidative stress, phosphorus metabolism, growth factors, and oxidative stress. Cellular components (CC) were mainly concentrated in receptor complexes, transcriptional regulatory complexes, and vesicle cavities. Molecular functions (MF) were remarkably rich in transcription factor binding and kinase binding (Figure 9B). KEGG pathway enrichment analysis showed the PD-1/PD-L1 signaling pathway played a certain role (Figure 9C). A Protein–Protein Interaction (PPI) network was constructed and key genes were screened. It was found that AKT, MAPK, FOXO, and SMAD were potential targets related to miRNA treatment of SCI, indicating these genes played important roles in regulating cell pathophysiological processes. They are key players in SCI treatment (Figures 9D,E).

## Discussion

### Research progress on the effects of miRNAs on SCI

Spinal cord injury is a nervous system disease causing physical and psychological harm to patients and a heavy economic burden on society (17). It can be divided into primary and secondary injury. Primary injury is typically mechanical, while secondary injury can lead to insufficient perfusion of gray matter post-injury and spread to surrounding white matter (18, 19). Injury-induced oxidative stress, inflammation, glial or fibrotic scar formation, demyelination, and nerve injury can result in partial paralysis (20). Current research focuses on neuroprotection, neurorepair, and scar inhibition therapy. Neuroprotective therapies focus on reducing/preventing secondary damage (6). The goal of nerve repair therapy is to reshape neurovascular recovery of the nervous system (21). Scar inhibition is a potential strategy to control scar growth and promote axonal regeneration by inhibiting astrocyte proliferation and hypertrophy (22, 23). Current clinical treatments mainly involve surgical decompression and drug therapy. Surgical decompression can improve spinal cord compression, increase blood flow at the injury site, and reduce expansion. However, surgical treatment can lead to greater trauma and can only provide symptomatic relief rather than a fundamental cure (24, 25). Injectable drug therapy can also cause immune rejection, as well as excessive drug release and rapid degradation (26). In the context of medical treatment, the central objective is to mitigate hindrances to axon growth, ensuring a microenvironment that is conducive to nerve development. As part of this, there is an ongoing exploration of strategies that could potentially rebuild damaged neural circuits. Simultaneously, there is a concerted effort to promote functional recovery, enhancing the overall quality of life for patients. Lastly, there is a continuous search for suitable grafts that could support and guide the process of axon regeneration, ensuring optimal outcomes, miRNA-based therapy, blood vessel intervention and combination of multiple treatments will play a key role in the treatment of SCI in the future (27).

MicroRNAs are considered important biomarkers and therapeutic targets in SCI development (28). They exhibit tissue specificity and stability; miRNA content changes post-SCI are prerequisites for biological effects (29). MiRNAs serve as pathological biomarkers for Alzheimer’s disease, epilepsy, brain injury, and other conditions (30, 31). Bioinformatics analyses show abnormal miRNAs’ potential target genes post-SCI involve SCI pathogenesis, including inflammation, oxidative stress, apoptosis, and neuronal death (32). They also impact astrocytes and scar formation (33). Research on miRNAs and SCI can inform SCI diagnosis and treatment, but miRNAs’ pathophysiological significance remains incompletely determined. We use various bibliometrics tools for quantitative analysis, citation analysis, and visualization to reveal research hotspots and prospects. Compared to traditional review and meta-analysis, our approach explores research trends across multiple dimensions, providing richer, deeper, and more diversified information. It offers new reference and direction for SCI treatment and development.

### General research trends on the role of miRNAs in SCI

Bibliometrics can systematically classify and analyze literatures on different topics, enabling better understanding of correlations between hot spots, trends, and topics within a research field (34). This study applies bibliometrics methods to examine literature on miRNAs’ role in SCI restoration. From 2008 to 2022, related literature rose yearly, totaling 754 articles. Publications remained high and entered rapid growth. Involving 50 countries/regions, 802 institutions, 278 journals, and 3,867 authors, indicating sustained researcher interest and a hot topic in SCI restoration.

China currently has clear advantages in terms of national paper count, citation count, institutional ranking, and author ranking. However, cooperative network analysis shows the United States occupies an important position in the cooperative network, with high H-index for research institutions, indicating the United States also plays an important role in promoting related work. China’s overall link strength in the cooperation network remains insufficient; international exchanges and cooperation should be strengthened. Ma, H and Feng, SQ from China are field leaders and may continue to hold leadership positions, but their research teams are primarily domestically based. Journal research shows European Review for Medical and Pharmacsciences is the most published journal, also with the highest H-index, while Experimental Neurology is the most cited. Considering journal division and impact factors, research quality in this field needs further improvement. Chinese research institutions and scholars can advance miRNA research in SCI recovery by strengthening international cooperation, introducing advanced methods and technologies, critically evaluating and improving research papers, and actively participating in academic conferences. Such efforts can enhance academic level and reputation, and contribute important scientific achievements to the field.

### Hotspots and research trends on the role of miRNAs in SCI

Cited and co-cited documents refer to research in which one document cites another, and multiple documents cite the same one, respectively (35). Research references help scholars understand hotspots and frontiers in a field, identify key documents with important value, reveal academic networks, and strengthen knowledge dissemination. Combining literature contents in Tables 5, 6, we found inflammation, neuron protection, axon regeneration, scar formation, and specific mechanisms post-SCI remain research foci.

Keyword analysis extracts keywords or key phrases through processing and statistically analyzing large document, article, or data sets, enabling understanding of research topics, hotspots, and basic content. This study’s keywords are spinal cord injury, expression, apoptosis, and miRNAs. Changes in miRNA gene expression lead to increased apoptosis and improved SCI. This strategy remains a hot research topic. miR-7a treats SCI by inhibiting neuronal apoptosis and oxidative stress (36), while miR-487b inhibits inflammation and neuronal apoptosis in SCI by targeting Ifitm3 (37). Based on keyword cluster analysis, nerve injury closely relates to inflammation. Currently, combined with keyword co-occurrence timeline and Reference citation bursts, keywords gradually transition to extracellular vesicles and neuroinflammation. Long et al. reported astrocyte-derived exosomes rich in miR-873a-5p inhibit neuroinflammation post-traumatic brain injury through microglial phenotypic regulation (38). Future directions include exosomes carrying miRNAs to inhibit inflammation for SCI treatment.

According to GO analysis and KEGG pathway, miRNAs actively participate in pathophysiological activities post-SCI. The PD-1/PD-L1 signaling pathway is involved in inhibiting neuroinflammation and improving traumatic brain injury (39). PPI network predicts AKT, MAPK, and FOXO as important pivotal genes, potentially key in apoptosis, autophagy, and inflammation. Currently, these genes are widely studied in SCI research. miR-21-5p, miR-92b-3p, and miR-34a all inhibit neuronal apoptosis and promote nerve regeneration via AKT (40–42). Excessive miR-340-5p reduces spinal cord injury-induced neuroinflammation and apoptosis by modulating P38-MAPK signaling (43). FOXO is widely reported in anti-inflammatory and anti-cancer studies (44, 45). SMAD plays an important role in SCI remyelination (46). Elucidating miRNA-target gene regulatory mechanisms may achieve reliable therapeutic effects and more comprehensive, effective rehabilitation programs for SCI patients.

### Prospects of therapeutic of miRNAs in SCI

At present, effective clinical treatment methods for SCI remain lacking, and emerging treatment methods such as cell transplantation, epigenetic regulation, artificial scaffold transplantation, and gene therapy are the main research directions for researchers (47, 48). Secondary injury usually leads to changes in the spinal cord microenvironment, resulting in changes in miRNA expression (49). This discovery provides potential opportunities for gene intervention (50). MiR-33-5p alleviates rat SCI and protects PC12 cells from lipopolysaccharide-induced apoptosis (51). MiR-433-5p overexpression protects against acute spinal cord injury by activating MAPK1 (52). MiR-7a improves spinal cord injury by inhibiting neuronal apoptosis and oxidative stress (36). Although the use of miRNAs as a therapeutic agent has attracted considerable attention due to its therapeutic effect on SCI, The limitations of using miRNAs as a therapeutic tool lie in their delivery and activation *in vivo* (29).

Exosomes, as intercellular communication carriers, play an irreplaceable role in maintaining multicellular organism functional integrity. Protected by a phospholipid layer, miRNAs can be accurately transported to target sites and avoid hydrolysis by extracellular enzymes, enhancing blood–brain barrier penetration and preventing misdelivery. Jiang et al. (53) confirmed neuron-derived exosome-delivered miR-124-3p protects spinal cord from traumatic injury by inhibiting neurotoxic microglia and astrocyte activation. Liu et al. (54) demonstrated hypoxic-preconditioned mesenchymal stem cell-derived exosome shuttle miR-216a-5p repaired traumatic spinal cord injury by altering microglia M1/M2 polarization. Recent miRNA delivery technology advances raise high expectations for miRNA therapeutics. While no clinical trials yet use exosome-attached miRNAs to treat human SCI, experimental data strongly support this approach’s feasibility. We must further analyze its beneficial effects and potential risks before applying it clinically.

## Limitations

This study, for the first time, conducted systematic bibliometric and visual analysis on miRNA and SCI-related documents extracted from the WOSCC database. Based on the Arrowsmith project, we also revealed potential mechanisms between miRNA and SCI. The study’s results laid a foundation for further research in this field. However, the study also has limitations. First, the included database data is relatively simple, and some other data may be ignored. Additionally, excluding articles not in English may impact conclusions. The database used for this study is updated in real-time and does not include papers published after the search date, which may result in differences between results at publication time and this result.

## Conclusion

The study highlights the global research interest, development trends and future clinical application potential of miRNAs in SCI treatment. We have confirmed the key role of miRNA in spinal cord injury and it has garnered continuous attention from researchers, becoming a hotspot of research in this field. Since 2008, annual publications in this field have increased. China leads in publication quantity, research institutions, and authors, but requires strengthened international collaboration. Current research focuses have shifted from single miRNA treatments to SCI treatment using exosome-attached miRNAs. In mechanism research, AKT, MAPK, FOXO, and SMAD are potential targets of miRNAs influencing SCI. There are relatively few clinical studies on miRNAs in SCI treatment. However, large-scale, multicenter clinical trials are needed to prove treatment effectiveness and safety.

## Data availability statement

The original contributions presented in the study are included in the article/supplementary material, further inquiries can be directed to the corresponding authors.

## Author contributions

BH: Methodology, Resources, Writing – original draft. YZ: Software, Visualization, Writing – original draft. CC: Conceptualization, Writing – original draft. BW: Investigation, Writing – original draft. HZ: Data curation, Writing – review & editing. BL: Supervision, Writing – review & editing. RZ: Validation, Writing – review & editing. FF: Validation, Writing – review & editing.

## Footnotes

^2^ https://bibliometric.com/

^3^ https://www.stringdb.org/
